# SC-III3, a novel scopoletin derivative, induces cytotoxicity in hepatocellular cancer cells through oxidative DNA damage and ataxia telangiectasia-mutated nuclear protein kinase activation

**DOI:** 10.1186/1471-2407-14-987

**Published:** 2014-12-19

**Authors:** Peng Zhao, Li Chen, Lin-Hu Li, Zhi-Feng Wei, Bei Tong, Yu-Gai Jia, Ling-Yi Kong, Yu-Feng Xia, Yue Dai

**Affiliations:** Department of Pharmacology of Chinese Materia Medica, China Pharmaceutical University, 24 Tong Jia Xiang, Nanjing, 210009 China; Department of Natural Medicinal Chemistry, China Pharmaceutical University, 24 Tong Jia Xiang, Nanjing, 210009 China; Department of Chinese Materia Medica Analysis, China Pharmaceutical University, 24 Tong Jia Xiang, Nanjing, 210009 China

**Keywords:** SC-III3, Hepatocellular cancer, ROS, DNA damage, Cell cycle arrest

## Abstract

**Background:**

Natural products from plants have been proven to be important resources of antitumor agents. In this study, we exploited the antitumor activity of (E)-3-(4-chlorophenyl)-N-(7-hydroxy-6-methoxy-2-oxo-2H-chromen-3-yl) acrylamide (SC-III3), a newly synthesized derivative of scopoletin, by *in vitro* and *in vivo* experiments.

**Methods:**

Human hepatocellular carcinoma cell line HepG2 cells and xenograft of HepG2 cells in BALB/c nude mice were used to investigate the effects of SC-III3 on hepatocellular cancers. Cell cycle arrest and apoptosis were analyzed by flow cytometry. Cell cycle arrest, apoptosis and ATM-Chk pathway-related proteins were characterized by western blot.

**Results:**

SC-III3 selectively inhibited the viability of HepG2 cells without significant cytotoxicity against human normal liver cells LO2. In mouse xenograft model of HepG2 cells, SC-III3 showed a marked inhibition of tumor growth in a dose-dependent manner. Cell cycle analysis revealed that SC-III3 induced cells to accumulate in S phase, which was accompanied by a marked decrease of the expressions of cyclin A, cyclin B, cyclin E and Cdk2 proteins, the crucial regulators of S phase cell cycle. SC-III3 treatment resulted in DNA breaks in HepG2 cells, which might contribute to its S phase arrest. The S arrest and the activation of ATM-Chk1/Chk2-Cdc25A-Cdk2 pathways induced by SC-III3 in HepG2 cells could be efficiently abrogated by pretreatments of either Ku55933 (an inhibitor of ATM) or UCN-01 (an inhibitor of Chk1/Chk2). The activation of p53-p21 pathway by SC-III3 was also reversed by Ku55933 treatment. SC-III3 led to significant accumulation of intracellular reactive oxygen species (ROS), a breaker of DNA strand, in HepG2 cells but not LO2 cells. Pretreatment with N-acetyl-l-cysteine (NAC), a ROS scavenger, could reverse SC-III3-caused ROS accumulation, DNA damage, activation of signal pathways relevant to DNA damage, S phase arrest and cell viability decrease in HepG2 cells.

**Conclusion:**

SC-III3 is able to efficiently inhibit the growth of hepatocellular carcinoma through inducing the generation of intracellular ROS, DNA damage and consequent S phase arrest, but lack of significant cytotoxicity against normal liver cells. This compound deserves further studies as a candidate of anticancer drugs.

## Background

Hepatocellular carcinoma (HCC) is the fifth most common solid tumor and the third largest cause of cancer-related death worldwide [1, 2]. Although a great achievement in HCC treatment has been acquired during the past three decades, patients still experience a poor prognosis because of the lack of effective therapeutic agents [3, 4]. Current therapeutic strategies for HCC, such as surgical resection, liver transplantation and a few of pharmaceutical interventions, fail to take an observable effect [5, 6]. Therefore, novel therapeutic strategies, including the utilization of new targeted agents, combination chemotherapy, and neoadjuvant therapy are needed urgently for the effective management of hepatocellular carcinoma.

Intracellular reactive oxygen species (ROS) is deeply involved in a great range of biological activities and disease states, such as cell fate-death or survival, cancer, diabetes, neurodegeneration and atherosclerosis [7]. At low to moderate levels, ROS may contribute to tumor formation either by acting as signaling molecules or by promoting the mutation of genomic DNA. For instance, ROS can stimulate the phosphorylations of p38 mitogen-activated protein kinase (MAPK), extracellular signal-regulated kinase (ERK) and JUN N-terminal kinase (JNK), which are beneficial for tumor cell growth and survival. However, at high levels, ROS will promote cell death and severe cellular damage such as DNA damage [8]. Furthermore, cancer cells have higher ROS levels than normal cells. This aspect provides a significant therapeutic window because cancer cells are more sensitive than normal cells to agents that induce further accumulation of ROS [9–11]. Therefore, perturbing ROS homeostasis is considered as a new strategy for cancer treatment.

In response to DNA damage caused by high levels of ROS, cell cycle checkpoints and a series effector kinases should be activated, which thereby regulates the cellular decision among cell cycle arrest, DNA repair, apoptosis or other cell death modalities [12]. Ataxia telangiectasia-mutated (ATM), ATM and RAD3-related (ATR) kinases are two members of the phosphoinositide 3-kinase-related protein kinase family that plays key role in DNA damage response. Following the activation of these kinases, the downstream effectors such as Chk1, Chk2 and p53 are activated [13, 14]. Then, activated Chk1 and Chk2 result in the phosphorylations of their downstream effectors such as Cdc25A, which regulate the activity of Cdk2 by dephosphorylation on Tyr15 [15, 16]. Cdk2-cyclinA complex, an important regulator of S-G2 phase cell cycle transition and the ultimate target of the checkpoint signaling pathway, is also inhibited by p21, the downstream effector of p53 [17].

Scopoletin (6-methoxy-7-hydroxycoumarin), a natural compound originating from *Erycibe obtusifolia* Benth and other plants, has been proven to possess a wide range of pharmacological properties, such as anti-angiogenic, anti-inflammatory, hypouricemic and anti-tumor activities [18–20]. It exerted antitumor effects on human prostate tumor cells and leukemia cells through inducing cell cycle arrest and triggering apoptosis [21, 22], and also showed considerable therapeutic potentials against 7, 12-dimethylbenz anthracene-induced skin cancer in mice [23]. Whereas, scopoletin has been demonstrated to exert far less profound effects *in vitro* and *in vivo*, and high *in vivo* elimination rate leads its effect to maintain only a few minutes. Moreover, recent studies have indicated that some derivatives of scopoletin could exhibit good antitumor effects *in vitro* and *in vivo* [24].

**Figure 1:**
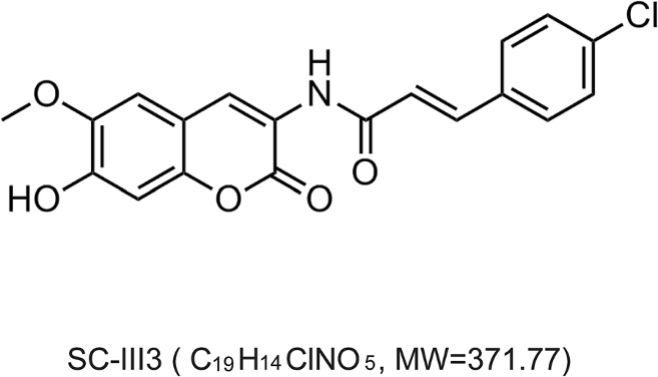
**The chemical structure of SC-III3.**

Recently, we synthesized a series of new scopoletin derivatives with different substituents. These derivatives were screened for *in vitro* cytotoxic activity against human cancer cell lines representing cancers of lung, colon, ovary as well as breast. Compared with doxorubicin, a standard potent anticancer drug, compound SC-III3 ((E)-3-(4-chlorophenyl)-N-(7-hydroxy-6-methoxy-2-oxo-2H-chromen-3-yl) acrylamide, Figure 1) showed potent anticancer activity at low concentrations against most of the used human tumor cell lines *in vitro*, and inhibition of the transplanted mouse lewis lung carcinomas *in vivo* [Li LH, Zhao P, Xia YF, Chen L: Synthesis, in vitro and in vivo antitumor activity of scopoletin-cinnamic acid hybrids, submitted]. In the present study, we investigated the antitumor effects of SC-III3 in hepatocellular carcinoma cells and a xenograft model of nude mice, and shed light on its possible mechanisms in views of oxidative DNA damage and cell cycle arrest.

## Methods

### Chemicals

SC-III3, (E)-3-(4-chlorophenyl)-N-(7-hydroxy-6-methoxy-2-oxo-2H-chromen-3-yl) acrylamide, was prepared by Dr. Chen Li (China Pharmaceutical University, China). The structure was identified by IR, ^1^H NMR, and HRMS. The purity was 99.51% determined with HPLC. It was applied in DMSO (Sigma-Aldrich, St. Louis, USA) to 10 mM and stored at -20°C. The concentrations used here were 0.03, 0.1, 0.3 and 1 μM for cellular treatment *in vitro* and freshly diluted with DMEM to final concentration. Cells in control groups were treated with the same amount of DMSO (0.01%) as used in the corresponding experiments. Doxorubicin was obtained from Shenzhen Main Luck Pharmaceuticals, Inc. (Shenzhen, China) and dissolved in phosphate buffered saline (PBS) to give a stock solution of 10 mmol/L. The solution was stored at -20°C, and kept away from light. MTT [3-(4, 5-dimethylthiazol-2-yl)-2, 5-dipheny-tetrazoliumbromide], N-acetyl-l-cysteine (NAC), Ku55933 and UCN-01 were obtained from Sigma-Aldrich (St. Louis, USA) and applied in 0.01 M PBS. Antibodies against p-ATM (Ser1981), ATM, p-ATR (Ser428), ATR, p-Chk1 (Ser345), p-Chk1 (Ser280), p-Chk1 (Ser296), Chk1, p-Cdk2 (Tyr15), p53, p21 were purchased from Cell Signaling Technology (Danvers, MA). p-Chk2 (Thr68), Chk2, Cdc25A, p-H2AX(Ser139), H2AX antibodies were purchased from EnoGene Biotech (Nanjing, China), GAPDH monoclonal antibodies were purchased from Kangchen Bio-tech (Shanghai, China); Cdk2, cyclinA, cyclinE, cyclinB, Bax, Bcl-2 monoclonal antibodies were purchased from Bioworld (Georgia, USA).

### Cell culture and animals

The human hepatoma HepG2 cell line was obtained from American Type Culture Collection (Bethesda, MD, USA). Cells were grown in DMEM medium (Gibco, USA) supplemented with 10% fetal bovine serum (Biological Industries, Israel), 100 units/mL penicillin and 60 μg/mL streptomycin (Gibco, USA) at 37°C in a humidified atmosphere comprised of 95% air and 5% CO_2_. Human normal liver cell LO2 was purchased from the Cell Bank of the Chinese Academy of Sciences (Shanghai, China). Cells were grown in RPMI-1640 medium (Gibco, USA) supplemented with 10% fetal bovine serum (Biological Industries, Israel). Exponentially growing culture was maintained in a humidified atmosphere comprised of 95% air and 5% CO_2_ at 37°C.

Specific pathogen-free male BALB/c nude mice, weighing 18–22 g, between 4 and 6 weeks of age, were purchased from Shanghai Slac Laboratory Animal Co. LTD (Shanghai, China). The mice were raised under controlled temperature (26-28°C, 55 ± 5% humidity) and daily light intensity (12 h of light), and were fed with standard laboratory food and water adlibitum. The animal experiments were conducted with the approval of the Animal Ethics Committee of China Pharmaceutical University.

### MTT assay

The HepG2 cells were initiated in 96-well microplate at a density of 5 × 10^3^ cells per well in 100 μL culture medium. The cells were incubated overnight and then treated with various concentrations of SC-III3 or doxorubicin, and cultured for 24 or 48 h. Subsequently, 20 μL/well of MTT solution (5 mg/mL) was added to each well. Plate was incubated at 37°C in a 5% CO_2_ atmosphere for 4 h, the supernatants were removed and 150 μL/well DMSO was added to dissolve formazan crystals. Plate was placed on an orbital shaker for 5 min, and the absorbance per well was recorded at 570 nm with a Model 1500 Multiskan spectrum microplate Reader (Thermo, Waltham, MA, USA). Data were analyzed from three independent experiments and then normalized to the absorbance of the wells containing media only (0%) and untreated cells (100%). IC50 value was taken as the concentration that caused 50% inhibition of cell viabilities and calculated by Graph pad Prism 5 software.

### Cell cycle analysis

HepG2 cells were treated with various concentrations of SC-III3 and were harvested by trypsinization, fixed with 70% ethanol and stored at -20°C for at least 1 day. Following fixation, cells were subjected to a PBS wash and then stained with DNA staining solution comprising 2.5 mg/mL propidium iodide (PI) and 50 mg/mL RNase A in PBS. Samples were incubated at 37°C for 30 min away from light and then analysed on a FACSCanto II flow cytometer (FACSCanto II, Becton Dickinson, USA).

### Apoptosis detection

HepG2 cells were treated with various concentrations of SC-III3 and were harvested by trypsinization and then washed twice with PBS. Cells were stained with Annexin V-FITC and PI, and loaded on a flow cytometer, set for FL1 (AnnexinV) and FL2 (PI) bivariate analysis Data acquisition and analysis were performed with a Becton–Dickinson FACSCalibur flow cytometer using Cell Quest software.

### Western blotting assay

HepG2 cells were treated with different concentrations of SC-III3 (0.03, 0.1, 0.3 and 1 μM) for 24 or 48 h. Subsequently, cells were washed twice with ice-cold PBS buffer (pH 7.2). Cells were lysed with lysis buffer (15 mM Tris [tris (hydro xymethyl) aminomethane] - HCl, 50 mM NaCl, 5 mM EGTA [ethyleneglycoltetraacetic acid], 1% TritonX-100, 1% sodium deoxycholate, 0.1% SDS [sodium dodecyl sulfate], 1 mM NaF, 1 mM PMSF [phenylmethylsulfonyl fluoride], 1 mM Na_3_VO_4_, 10 μg/mL aprotinin, and 10 μg/mL leupeptin, pH 7.4) and centrifuged. The concentration of total proteins was measured by Bradford assay with a Varioskan spectrofluorometer and spectrophotometer (Thermo, Waltham, Massachusetts) at 595 nm. Samples were fractionated on a 10% SDS-PAGE, stacked at 80 V for 35 min and separated at 120 V for 1 h and transferred to PVDF membranes at 15 V. Membranes were blocked for 1 h at room temperature with 5% nonfat-milk and then incubated with different primary antibodies overnight at 4°C. Then, they were incubated with secondary antibodies for 1 h at 37°C. The bands were visualized using film exposure with ECL reagent.

### Measurement of ROS level

The production of cellular reactive oxygen species (ROS) was detected using fluorescent dye 2, 7-dichlorofluorescein-diacetate (DCFH-DA, Beyotime Institute of Biotechnology, China) sensitively as previously described [25]. In brief, the HepG2 cells were pretreated with/without NAC for 2 h and then exposed to SC-III3 for 12 h. Then cells were collected and incubated with 10 μM DCFH-DA attenuated with serum-free medium for 30 min at 37°C in the dark. After washed by serum-free, ROS level was measured by the intensity of the fluorescence on a Model 1500 Multiskan spectrum microplate Reader (Thermo, Waltham, MA, USA).

### Animal study

After acclimatized to their new environment for 1 week, the mice were injected subcutaneously with HepG2 cells (4 × 10^6^/0.2 mL/mouse) into the right flank. When tumors reached a mean group size of approximately 100 mm^3^, the mice were randomly divided into five groups with 10 mice each (day 0). Then mice were treated by intraperitoneal injection of solvent (0.5% CMC-Na), SC-III3 (2.5, 5 and 10 mg/kg) or doxorubicine (2.5 mg/kg) every day for 2 weeks. Tumor volume was measured every 3 days. The tumor volume was calculated by the formula 1/2 × larger Diameter × (smaller diameter)^2^. At the 15th day, all mice were killed and the tumors were segregated, weighed and stored in -80°C. Tumor inhibitory ratio was calculated by the following formula: Tumor inhibitory ratio (%) = [(W_control_ - W_treated_)/W_control_] × 100%. W_treated_ and W_control_ were the average tumor weight of the treated and control group, respectively [26].

### Statistical analysis

For analysis of data, values were presented as mean ± SEM for three independent experiments. The differences between the groups were examined for statistical significance using non-paired Student’s two-tailed *t* test with Prism 5 software.

## Results

### SC-III3 inhibited the growth of human liver cancer cells *in vitro*and *in vivo*

**Figure 2:**
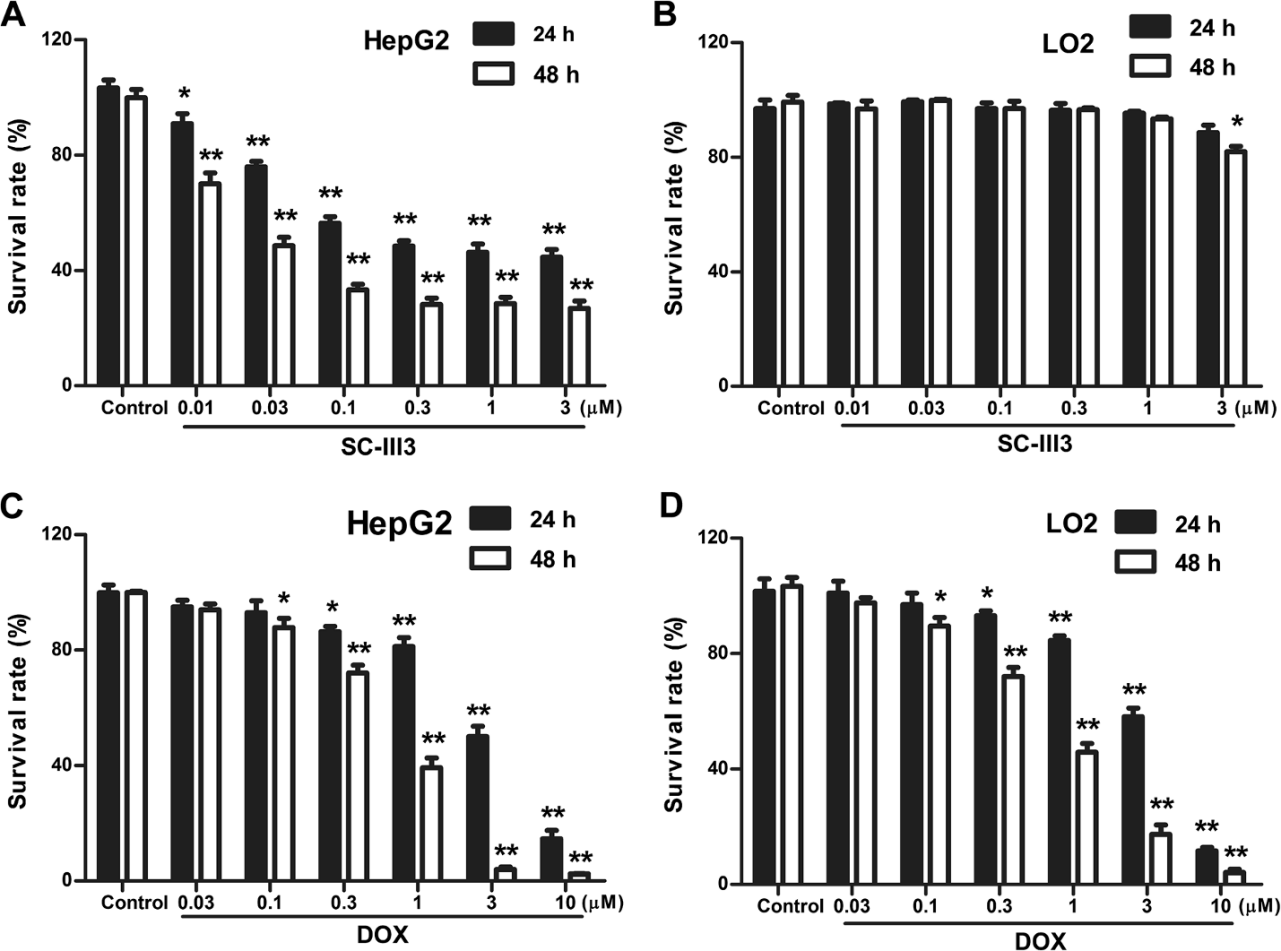
**SC-III3 inhibited cell growth of HepG2 cells. (A)** HepG2 cells were treated with SC-III3 for 24 and 48 h, and cell viability was analyzed by MTT assay. **(B)** LO2 cells were treated with SC-III3 for 24 and 48 h. **(C)** HepG2 cells were treated with doxorubicin (DOX) for 24 and 48 h. **(D)** LO2 cells were treated with DOX for 24 and 48 h. Data were presented as means ± SEM of three separate experiments. The difference were significant at *p < 0.05, **p < 0.01 *vs.* control.

At first, we examined the effect of SC-III3 at different concentrations on cell viability. As shown in Figure 2A, after 24 h and 48 h of treatment, SC-III3 obviously reduced the number of viable cells, and the IC_50_ values were 0.65 and 0.32 μM, respectively. In contrast, a normal liver cell line LO2 was less sensitive to SC-III3 (Figure 2B). HepG2 and control LO2 cells were also treated with increasing concentrations of doxorubicin (DOX), a broad-spectrum chemotherapeutic drug, for 24 and 48 h. DOX caused a concentration-dependent decrease in HepG2 cell viability with an IC_50_ value of 3.1 μM at 24 h and 1.2 μM at 48 h (Figure 2C). Meanwhile, DOX also significantly decreased LO2 cell ability with an IC_50_ of 3.5 μM at 24 h and 0.92 μM at 48 h (Figure 2D). Our data showed that SC-III3 significantly suppressed the proliferation of liver cancer cells within a 48 h period, but it had no effect on normal liver cells distinct from DOX.

**Figure 3:**
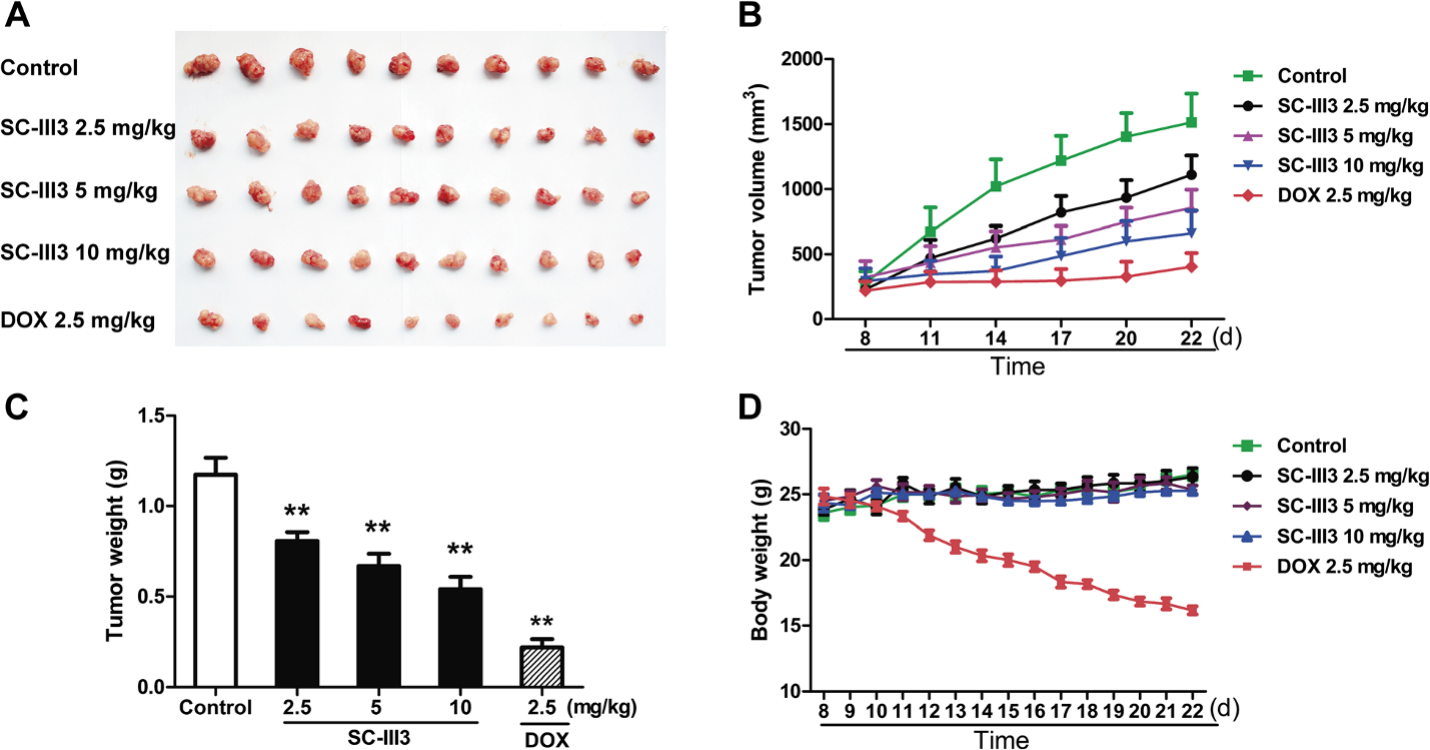
**SC-III3 markedly inhibited tumor growth in vivo.** Mice were intraperitoneally injected with 2.5, 5 and 10 mg/kg of SC-III3 once a day for 14 days. Control was treated with normal vehicle, and positive control was intraperitoneally injected with 2.5 mg/kg of doxorubicin. **(A)** Resected tumor images from the experimental. **(B)** Tumor volume was measured every 3 days. **(C)** Tumor mass were weighed. **(D)** Nude mice weight was recorded once a day. Data were presented as Mean ± SEM, n = 10. The difference were significant at *p < 0.05, **p < 0.01 *vs.* control.

The ability of SC-III3 in killing HepG2 cells *in vitro* led us to evaluate its anti-tumor activity *in vivo*. A xenograft mouse model of HepG2 cells was therefore established (Figure 3A). The results showed that SC-III3 significantly inhibited tumor growth during the 14-day experimental period in a dose-dependent manner (Figure 3B). As shown in Figure 3C and 3D, compared with the control group, SC-III3 significantly reduced the tumor weight. And the inhibition rates caused by SC-III3 (2.5, 5 and 10 mg/kg) were 31.2, 38.6, and 52.4%, respectively. Meanwhile, mouse body weights were lack of significant difference between SC-III3-treated and control groups during the experiment. The tumor growth inhibition rate of DOX group was 81.5%. However, DOX treatment significantly reduced the mouse body weight. These data demonstrated that SC-III3 inhibited tumor growth of HepG2 xenograft mouse model in a dose-dependent manner without significant side effect.

### SC-III3 induced S cell cycle arrest in liver cancer cells

**Figure 4:**
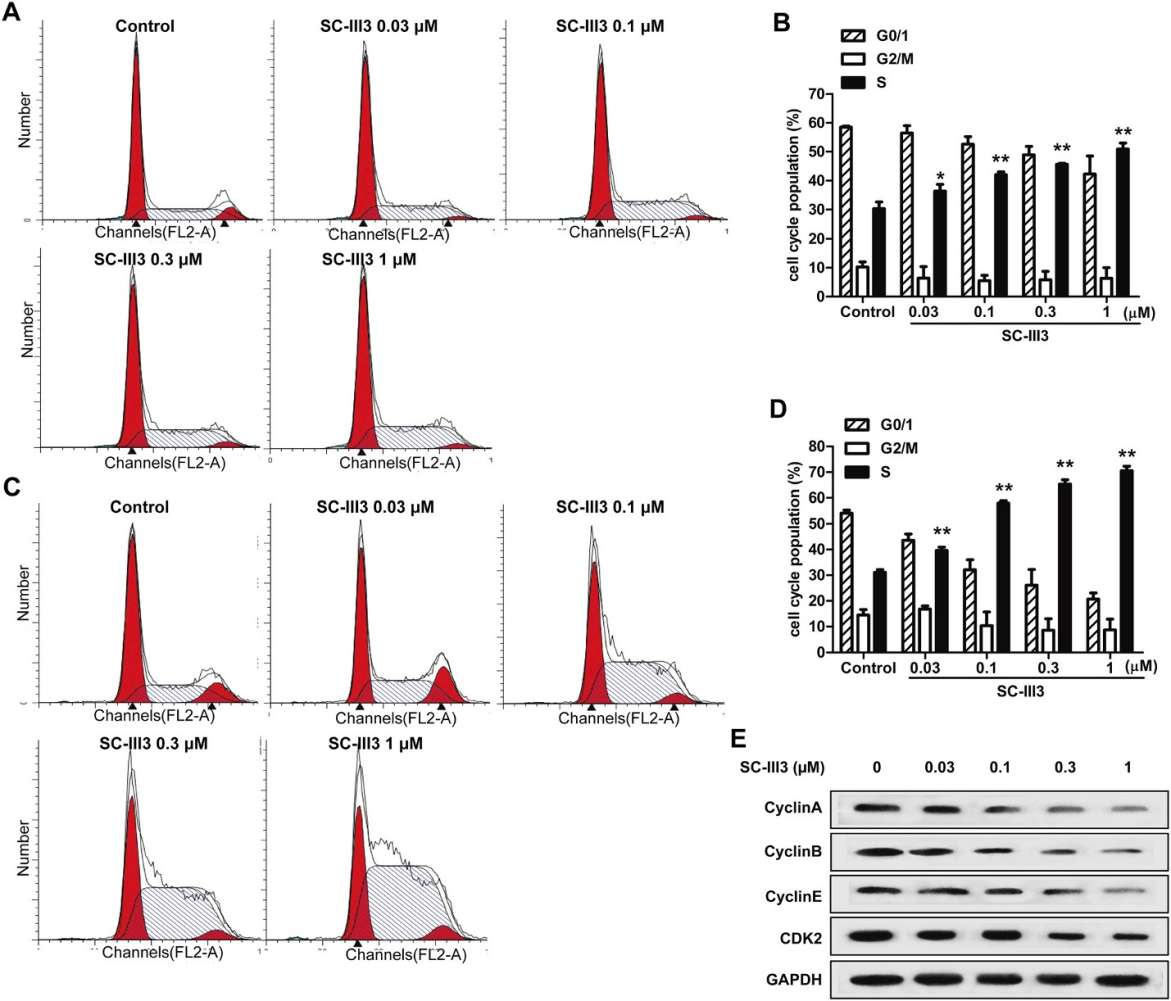
**SC-III3 induced HepG2 cell cycle arrest. (A, B)** The cells were treated with SC-III3 for 24 h, and the cell cycle was tested by flow cytometry after staining with propidium iodide. Cells in G0/G1, S, and G2/M phases were quantified and presented. **(C, D)** The cells were treated with SC-III3 for 48 h. **(E)** HepG2 cells were treated with SC-III3 for 24 h. The protein levels of Cyclin A, Cyclin B, Cyclin E and Cdk2 were analyzed with western blotting. Data were presented as means ± SEM of three separate experiments. The difference were significant at *p < 0.05, **p < 0.01 *vs.* control.

Whether the antiproliferative activities of SC-III3 was due to the induction of cell cycle arrest was addressed. After treated with various concentrations of SC-III3 for 24 and 48 h, respectively, HepG2 cells were fixed. Then cell cycle populations were determined by flow cytometry. As shown in Figure 4, after SC-III3 (0.03, 0.1, 0.3, 1 μM) treatment for 24 h, the cell percentages at S phase were increased from 30.3 ± 3.1% to 34.3 ± 3.3%, 42.1 ± 2.4%, 45.6 ± 1.5%, and 50.9 ± 2.9%, respectively (Figure 4A, B); For 48 h, the cell percentage at S phase were increased from 31.1 ± 1.5% to 38.6 ± 3.2%, 57.9 ± 1.3%, 65.3 ± 2.3%, and 70.6 ± 2.4%, respectively (Figure 4C, D). The results showed that SC-III3 induced a time- and concentration-dependent S phase arrest significantly. Western blot assay indicated that SC-III3 treatment for 24 h resulted in a concentration-dependent reduction of the expressions of cyclin A, cyclin B, cyclin E and Cdk2 (S cell cycle regulators) in HepG2 cells (Figure 4E). These studies suggested that the inhibition of cell growth by SC-III3 is associated with the induction of S phase arrest.

### SC-III3 caused apoptosis of liver cancer cells

**Figure 5:**
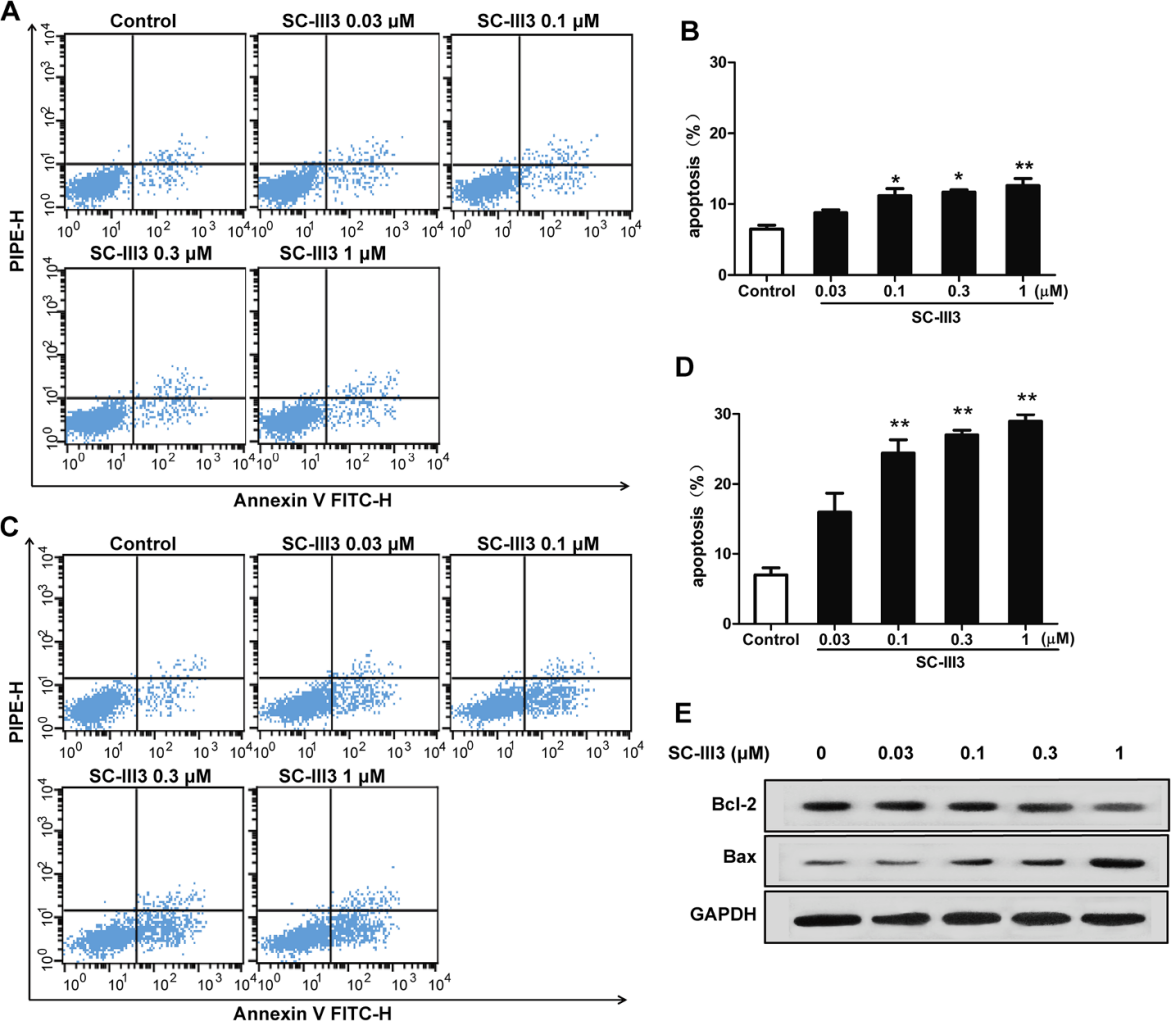
**SC-III3 induced HepG2 apoptosis in HepG2 cells. (A, B)** Cells were treated with SC-III3 for 24 h. Annexin V/PI double-staining assay of HepG2 was analyzed by flow cytometry. The percentages of cells in apoptosis were represented. **(C, D)** Cells were treated with SC-III3 for 48 h. **(E)** HepG2 cells were treated with SC-III3 for 48 h. The protein levels of Bcl-2 and Bax were analyzed with western blotting. Data were presented as means ± SEM of three separate experiments. The difference were significant at *p < 0.05, **p < 0.01 *vs.* control.

On the basis of the S phase arrest caused by SC-III3, we further examined the apoptosis rate of HepG2 cells by flow cytometry analysis. As shown in Figure 5, after treated with SC-III3 (1 μM) for 24 h, the apoptotic rate of HepG2 cells was less than 15% (Figure 5A, B). While exposed to SC-III3 (0.03, 0.1, 0.3 and 1 μM) for 48 h, the apoptotic percentages of HepG2 cells were increased to 15.9 ± 3.8%, 24.4 ± 2.6%, 27.1 ± 1.4%, and 28.9 ± 1.3%, respectively (Figure 5C, D). Then we further evaluated the levels of apoptosis-related proteins in HepG2 cells treated with SC-III3 for 48 h. As shown in Figure 5E, SC-III3 could reduce the expression of Bcl-2 but concomitantly increase the expression of Bax. It was suggested that SC-III3 might mainly interfere with the cell cycle of HepG2 cells, which in turn led to slight cell apoptosis.

### SC-III3 induced DNA damage response and the activation of ATM/Chk pathway

**Figure 6:**
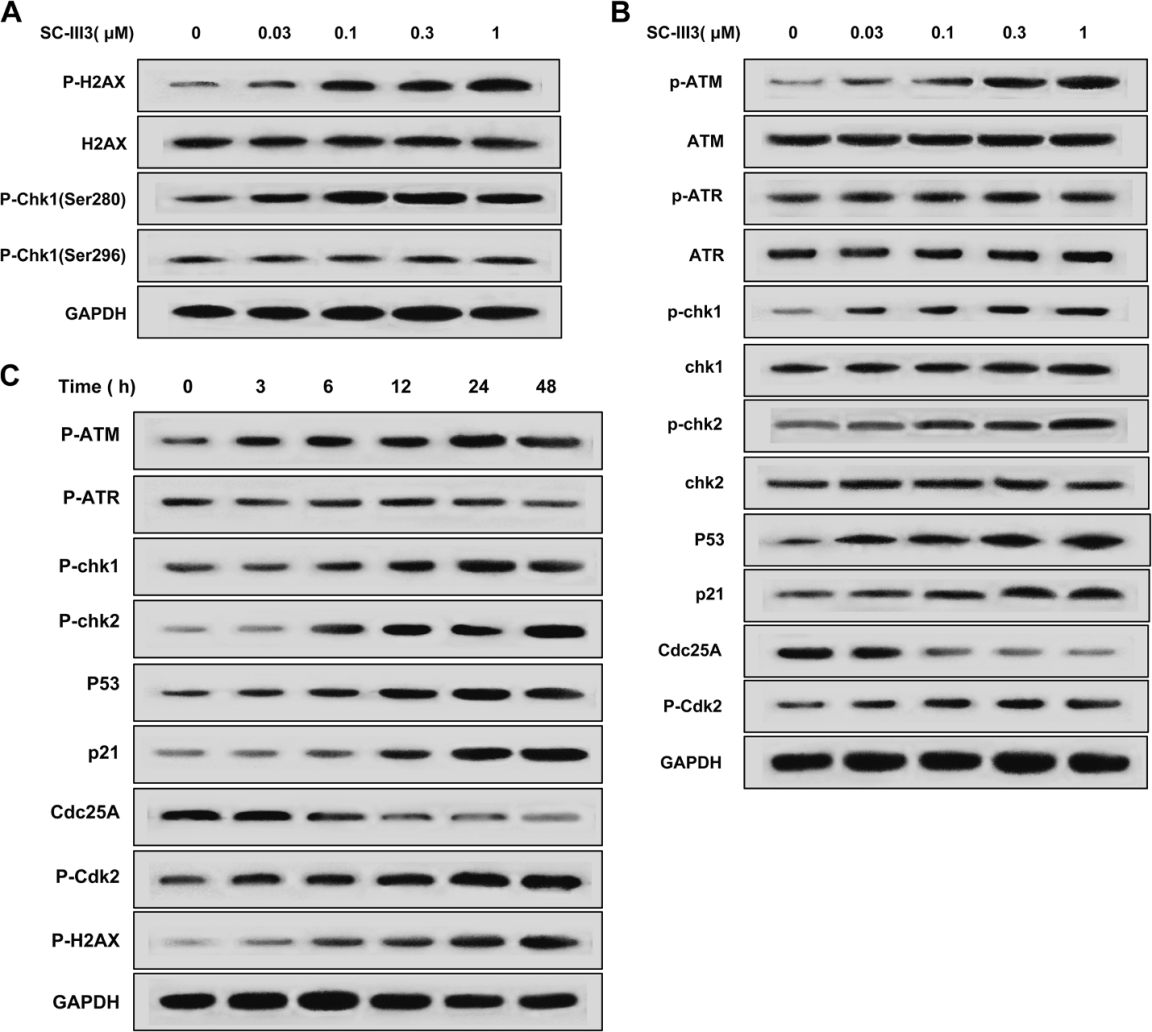
**Effect of SC-III3 on DNA damage and ATM/ATR pathways. (A)** HepG2 cells were treated with SC-III3 for 24 hours. Western blot analysis of p-H2AX, total H2AX, p-Chk1 (Ser280) and p-Chk1 (Ser296) response to SC-III3. **(B)** HepG2 cells were treated with SC-III3 for 24 hours. Western blot analyses of the ATM/ATR signaling related proteins in HepG2 cells. **(C)** HepG2 cells were treated with 1 μM SC-III3 for different times as indicated. Western blot analyses of p-ATM, p-ATR, p-Chk1, p-Chk2, Cdc25C, p-CDK2, p53, and p21 in HepG2 cells. These experiments were done in triplicates.

Because DNA damage was strongly correlated with the progression of cells through the S phase of the cell cycle [12], we assumed that SC-III3-induced S-phase arrest of HepG2 cells was due to the damage of DNA. To verify this point, we investigated the phosphorylations of histone variant H2AX at Ser-139 and Chk1 at both Ser-280 and Ser-296, the indicators of DNA breaks. SC-III3 markedly increased the levels of phosphorylated H2AX at Ser-139 and Chk1 at Ser-280 in a concentration-dependent manner, whereas did not affect the phosphorylation of Chk1 at Ser-296. These results indicated that SC-III3 substantially led to DNA damage in HepG2 cells (Figure 6A).

Among DNA damage response network, ATM and ATR play central roles [13, 14]. The regulation of Chk1/2 and p53 function is the best known pathway by which ATM and ATR regulate the cell cycle. The changes of key downstream effector proteins of ATM/ATR signaling, including ATM, ATR, Chk1, Chk2, Cdc25A and p53, in response to SC-III3 were investigated. As shown in Figure 6B, exposing HepG2 cells to SC-III3 (0.03, 0.1, 0.3 and 1 μM) for 24 h significantly increased the phosphorylation levels of ATM at Ser1981, Chk1 at Ser345, Chk2 at Thr68 and Cdk2 at Tyr15, increased the protein levels of p53, p21 and decreased Cdc25A, while did not affect the protein levels of ATM, ATR, Chk1 and Chk2 and the phosphorylation levels of ATR at Ser428.

Time-course for SC-III3 (1 μM)-induced activation of ATM pathway of HepG2 cells showed that SC-III3 increased the phosphorylation level of ATM (Ser1981) at 3 h, which persisted up to 24 h, then slightly declined at 48 h. The phosphorylations of Chk1 (Ser345) and Chk2 (Thr68) were also increased by SC-III3 treatment for 6 h, and maintained the elevated levels up to 24 h. The Cdc25A, a phosphatase that plays a critical role in G1-S transition and S-phase progression, was down-regulated at 6 h after SC-III3 exposure in HepG2 cells. This modulation was consistent with the activation of Chk1 and Chk2. The protein levels of p53 and p21 were increased at 6 and 12 h, respectively. Meanwhile, SC-III3 induced slightly accumulation of phospho-Cdk2 (Tyr15) at 12 h, which persisted up to 48 h in HepG2 cells (Figure 6C). These results suggested that the activation of ATM-Chk1/Chk2-Cdc25A-Cdk2 and p53-p21 pathways are involved in the effect of SC-III3.

### Inhibitors of ATM and Chk1/Chk2 reversed the S phase arrest by SC-III3

**Figure 7:**
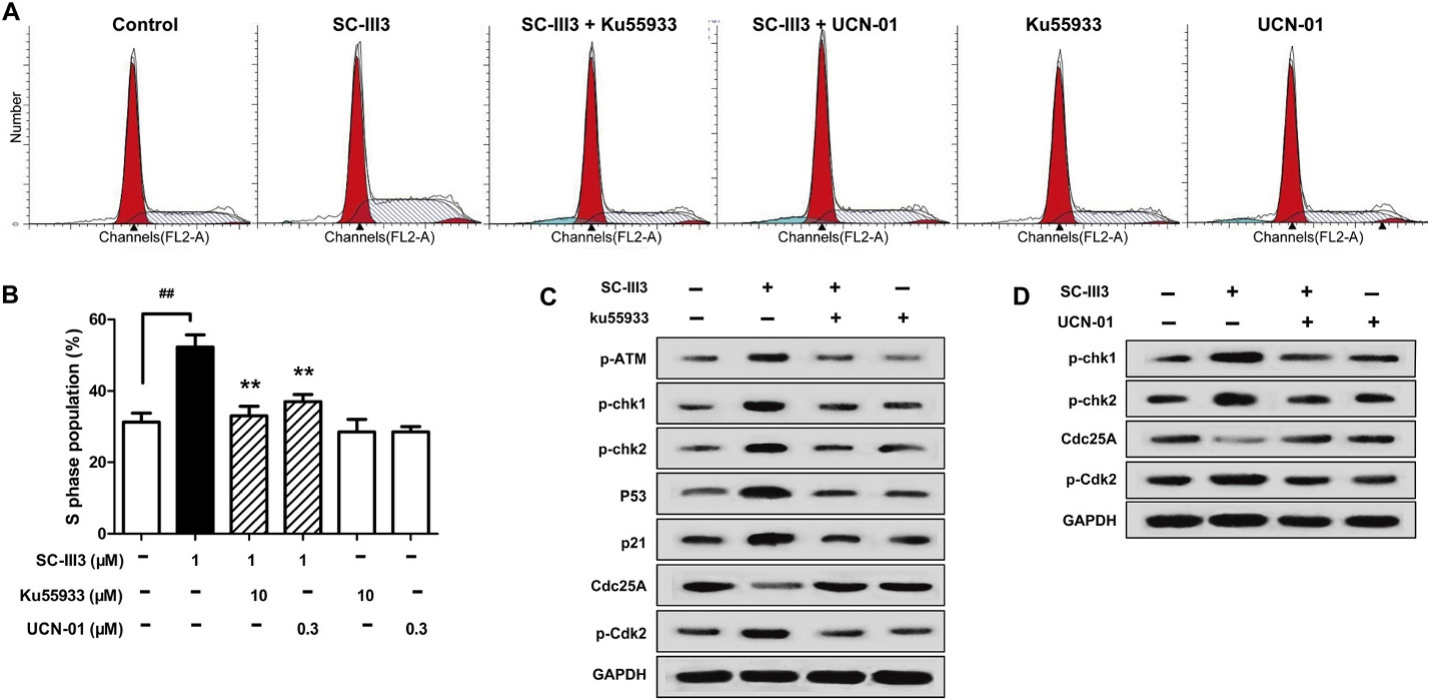
**Role of ATM/Chk1/Chk2 in SC-III3-mediated S cell cycle arrest. (A, B)** HepG2 cells were exposed to 1 μM SC-III3 for 24 h with or without adding of 10 μM Ku55933 or 0.3 μM UCN-01. Then, cells were analyzed for cell cycle distribution by flow cytometry. Cells in G0/G1, S, and G2/M phases were quantified and presented. **(C)** HepG2 cells were exposed to 1 μM SC-III3 with or without adding of 10 μM Ku55933 for 24 h. Then, western blot analyses of p-ATM, p-ATR, p-Chk1, p-Chk2, Cdc25C, p-CDK2, p53, and p21 in HepG2 cells. **(D)** HepG2 cells were exposed to 1 μM SC-III3 with or without adding of 0.3 μM UCN-01 for 24 h. Then, western blot analyses of p-Chk1, p-Chk2, p-Cdk2, and Cdc25A in HepG2 cells. The Data were presented as mean ± SEM for three separate experiments. The difference were significant at **p < 0.01 compared with SC-III3 (1 μM) and ^##^ *p* < 0.01 compared with SC-III3 (0 μM).

To gain insight into the relative contribution of ATM-Chk1/Chk2 pathway in SC-III3-mediated S phase arrest, Ku55933 (an inhibitor of ATM) and UCN-01 (an inhibitor of Chk1 and Chk2) were adopted, respectively [27, 28]. As shown in Figure 7A and 7B, Ku55933 treatment completely abrogated SC-III3-induced S phase arrest. The effect of Ku55933 on the activation of Chk1/2-Cdc25A-Cdk2 and p53-p21 pathway in response to SC-III3 treatment was further investigated. It was shown that Ku55933 could suppress the phosphorylations of ATM on Ser1981, Chk1 on Ser345, Chk2 on Thr68, and Cdk2 on Tyr15 induced by SC-III3. Meanwhile, SC-III3-mediated expressions of p53 and p21 (downstream effectors of ATM) were down-regulated by Ku55933. Furthermore, the down-regulated expression of Cdc25A by SC-III3 was also reversed by Ku55933 (Figure 7C). Similar to Ku55933, UCN-01 also dramatically reversed the S arrest of HepG2 cells by SC-III3 (Figure 7A, B). Furthermore, compared with SC-III3-treated group, a dramatic increase of Cdc25A and decreased phosphorylations of Chk1 on Ser345, Chk2 on Thr68, Cdk2 on Tyr15 were observed in the group co-treated with UCN-01 and SC-III3 (Figure 7D). Together, these data demonstrated that ATM-Chk1/Chk2 and their downstream signals were rapidly activated in HepG2 cells by SC-III3 exposure, and these signals might play critical roles in SC-III3-induced S arrest.

### SC-III3 triggered ROS generation

**Figure 8:**
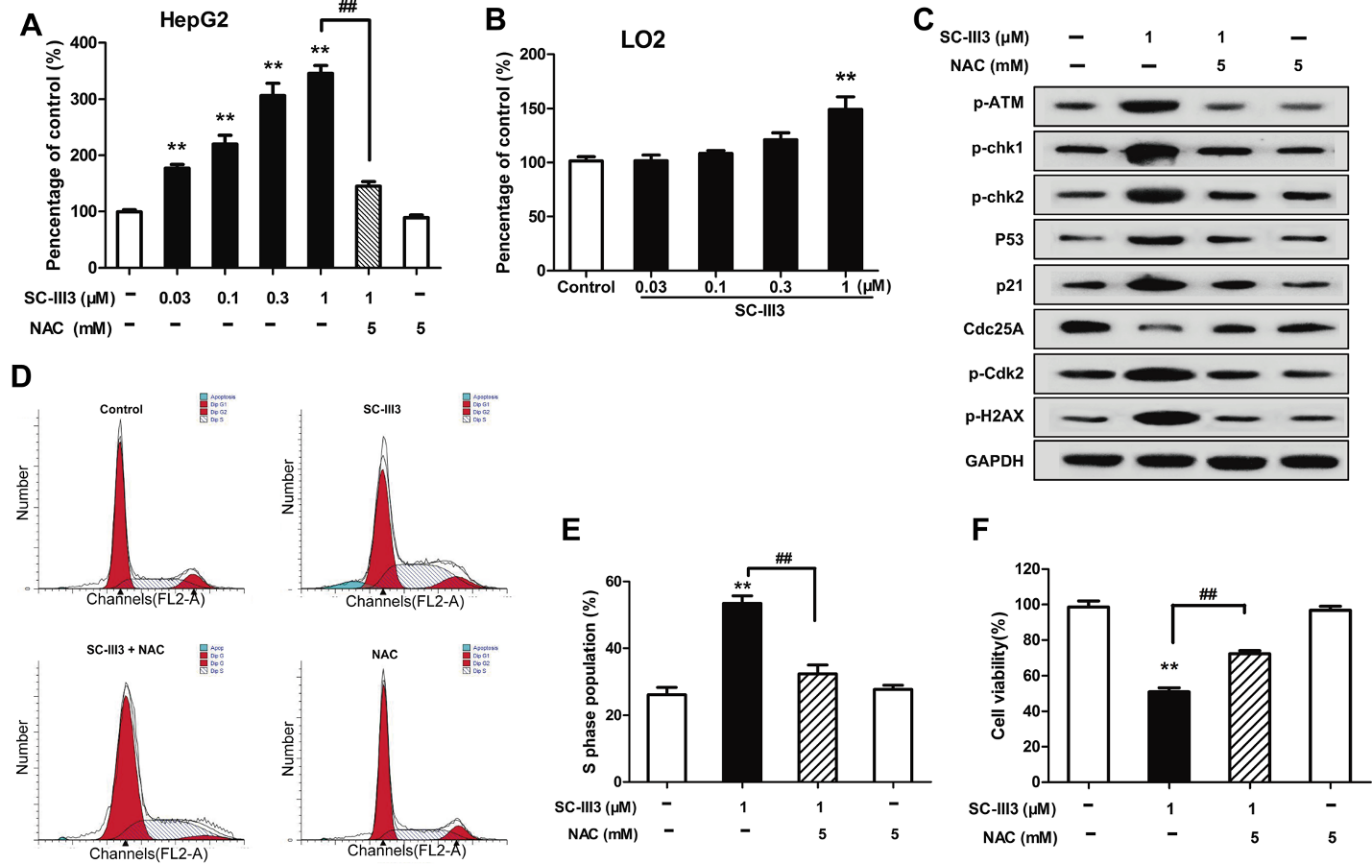
**SC-III3 induced DNA damage by activation of ATM pathway** ***via*** **ROS generation. (A)** HepG2 cells were pretreated with/without NAC for 2 h and then exposed to SC-III3 for 12 h. ROS level was determined with fluorescence probe DCFH2-DA. **(B)** LO2 cells were exposed to SC-III3 for 12 h. **(C)** HepG2 cells were pretreated with/without NAC for 2 h and then exposed to SC-III3 for 24 h. Then, cell lysates were prepared for Western blotting. **(D, E)** HepG2 cells were pretreated with/without NAC for 2 h and then exposed to SC-III3 for 24 h. Then, cells were analyzed for cell cycle distribution by flow cytometry. Cells in G0/G1, S, and G2/M phases were quantified and presented. **(F)** HepG2 cells were pretreated with/without NAC for 2 h and then exposed to SC-III3 for 24 h. Then, cell viability was analyzed by MTT assay. Data were presented as mean ± SEM for three separate experiments. The difference were significant at **p < 0.01 compared with SC-III3 (1 μM) and ^##^ *p* < 0.01 compared with SC-III3 (0 μM).

High levels of ROS can induce DNA damage, cell cycle arrest and apoptosis [9, 29]. To investigate the effect of SC-III3 on ROS generation, we detected the levels of ROS in SC-III3-treated cells. It was shown that SC-III3 induced a substantial increase in ROS levels in HepG2 cells. In addition, NAC, a ROS scavenger, significantly reduced the accumulation of ROS induced by SC-III3 (Figure 8A). In contrast, SC-III3 did not cause an significant increase in ROS levels in normal cells LO2 (Figure 8B). To further investigate whether the generation of ROS was a crucial step in SC-III3-induced DNA break, ATM-Chk pathway activation and S phase arrest, HepG2 cells were pretreated with/without NAC and then exposed to SC-III3. NAC pretreatment almost completely abrogated the ability of SC-III3 to promote the phosphorylation of H2AX, a well-known indicator in response to DNA damage, and in activating ATM-Chk1/Chk2-Cdc25A and p53-p21 pathways (Figure 8C). Furthermore, SC-III3-caused cell cycle arrest and cell viability decrease were also significantly blocked by NAC (Figure 8D, E, F). These results indicated that ROS mediated the DNA damage induced by SC-III3, and ROS activated S phase checkpoint through ATM-activated Chk1/Chk2-Cdc25A and p53-p21 pathway.

## Discussion

Hepatocellular carcinoma (HCC), which has highly chemical-resistant to currently available chemotherapeutic agents, is one of the most common causes of cancer mortality [30]. Traditional Chinese medicines have been recognized as a new source of anticancer drugs as new chemotherapy adjuvant by enhancing the efficacy and diminishing the side effects of chemotherapies. Scopoletin is the main active constituent of *E. obtusifolia* Benth stems and exerts anti-inflammation, anti-angiogenesis and anti-cancer effects [18, 19, 21–24]. We modified the structure of scopoletin and finally obtained a new compound, named SC-III3. In this study, SC-III3 showed significant inhibitory effect on the growth of HepG2 cells *in vitro* and the HepG2 xenografts *in vivo*. Then, we dissected the fundamental mechanisms of SC-III3 fighting against hepatocellular carcinoma: SC-III3 induced ROS-mediated DNA damage, which activated S phase checkpoint through ATM-activated Chk1/2-Cdc25A-Cdk2 and p53-p21 cascade.

SC-III3 could significantly inhibit HepG2 cell proliferation and growth in a concentration- and time-dependent manner. Low concentrations (0.03-1 μM) of SC-III3 showed significantly inhibitory effect on HepG2 cell proliferation after 24 h and 48 h treatment, which clearly demonstrated that SC-III3 was a potent cytotoxic compound. But this inhibitory effect is selective, exhibiting very little effect on the growth of LO2 cell, a normal liver cell line. In our studies, SC-III3 showed inhibitory effect on the hepatocellular carcinoma cells xenograft in nude mice. SC-III3 treatment in nude mice bearing tumors resulted in a significant inhibition of tumor volume without any apparent sign of toxicity. DOX administration also significantly inhibited the tumor growth of HepG2 xenograft, but caused serious side effects to the mice.

Cancer is considered a disease of the cell cycle. This assessment is supported by the fact that normal cells only proliferate in response to growth stimuli and specific mitogenic signals, but cancer cells proliferate in an unregulated way. Moreover, new links between alterations in the cell cycle regulatory machinery and tumorigenesis are being constantly reported and virtually all molecular species involved in regulating cell proliferation described in the literature have been related to malignant transformation [31]. Therefore, we assumed that SC-III3-induced cell growth inhibition was due to the cell cycle arrest. In the present study, we found that SC-III3 induced dose-dependent S-phase cell cycle arrest in HepG2 cells (Figure 3A). Cycle progression is controlled by several cyclin-dependent kinases (Cdks) and their cyclin partners. A number of Cdks regulate the cell cycle event in mammalian cells. Among the Cdks that regulate cell cycle progression, Cdk2 kinases is activated primarily in association with cyclin A, cyclin B and cyclin E in the S-phase progression [30–33]. In this study, cell cycle analysis revealed a prominent S-phase arrest of SC-III3-treated cells, and Cdk2, cyclin A, cyclin B and cyclin E proteins were down-regulated by SC-III3 treatment (Figure 4).

In advanced HCC, cancer cells usually become resistant to apoptosis [5, 6]. Thus, identification of SC-III3 that can induce apoptosis in HCC cells is highly desirable. The results demonstrated that treatment of HepG2 cells with SC-III3 for 48 h led to an elevated expression of Bax and reduced expression of Bcl-2, and then increased the ratio of Bax/Bcl-2, which contributed to SC-III3-induced apoptosis of HepG2 cells.

Treatment with various concentrations of SC-III3 for 24, 48 h significantly induced S phase arrest in HepG2 cells at early time point (24 h). In contrast, flow cytometry analysis revealed that treating HepG2 cells with 1 μM of SC-III3 for 24 h resulted in only a slight increase in apoptosis, and treating HepG2 cells with 1 μM of SC-III3 for 48 h led to a moderate increase in apoptosis. Such findings suggest that SC-III3 mainly suppressed the HepG2 cell cycle, which in turn led to induction of cell apoptosis.

We continued to investigate the detailed molecular mechanism of SC-III3-induced S cell cycle arrest and apoptosis in HepG2 cells. Many drugs in use disrupt genome integrity by causing DNA strand breaks, and consequently block cell proliferation mainly by inhibiting factors that enable cells to proceed from one cell cycle phase to the next through checkpoints in the cell division cycle [34]. ATM and ATR play key roles in DNA damage response. Generally, ATM is primarily activated in response to DNA strand breaks, such as those induced by ionizing radiation, whereas ATR is predominantly activated in response to damage caused by UV rays or replication block. The downstream effectors such as Chk1, Chk2 and p53 were activated by ATM and ATR [13, 14]. For instance, Chk2 was activated by phosphorylation at Thr68 by ATM, while Chk1 activation requires phosphorylation at Ser317 and Ser345 catalyzed by ATM and/or ATR. Furthermore, the activated Chk1 and Chk2 phosphorylate their downstream effectors such as Cdc25A which regulate the activity of the Cdk2 by dephosphorylating on Tyr15. Although ATM primarily phosphorylates the Chk2, while ATR preferentially phosphorylates the Chk1, accumulating evidences indicated that a cross-talk occurs between the two pathways [15–17]. Cdk2-cyclinA complexes, critical S phase regulator, were inhibited by p21, the downstream effector of p53. Then, we hypothesized that SC-III3 induced DNA damage, leading to S cell cycle arrest and apoptosis responses. The data demonstrated that SC-III3 induced DNA damage in HepG2 cells in a concentration- and time-dependent manners as confirmed by the upregulated expression of p-H2AX (Ser-139) and p-Chk1 (Ser-280), the biomarker for DNA damage. After the generation of DNA damage, ATM, the main sensor of DNA damage, was activated by phosphorylation at its serine 1981 in SC-III3-treated cells. The ATM inhibitor, Ku55933, reversed the S arrest and ATM-Chk1/Chk2-Cdc25A-Cdk2 and p53-p21 activation triggered by SC-III3, the results showed a causal link between ATM activation and S arrest in the treated cancer cells. Because both the checkpoint kinases Chk1 and Chk2 are the immediate substrates of ATM [35], our data showed that ATM significantly activates them by phosphorylation. Inhibiting Chk1/Chk2 by UCN-01 could abolish SC-III3-induced S phase arrest and S checkpoint, which indicated that these two kinases also contribute to the S arrest induced by SC-III3 in HepG2 cells. Thus, our data reveal the mechanism whereby SC-III3 induces S arrest and the existence of a mechanistic link between the SC-III3-caused DNA damage signals and the activation of the ATM-Chk1/2 pathway leading to the S arrest.

It is known that the therapeutic effect of many anticancer drugs is related to generation of intracellular ROS. Furthermore, ROS induced oxidative DNA damage has been well established. In normal cells, ROS are at low levels, which is pivotal for the modulation of normal cell proliferation. While, tumor cells, with a higher level of ROS close to the threshold of cytotoxicity, are susceptible to ROS-generating agents [9, 36, 37]. In this study, we found that SC-III3 caused a marked increase in ROS levels in HepG2 cells, which could be almost completely suppressed by NAC. In contrast to the results in HepG2 cells, SC-III3 did not cause an significant increase in ROS levels in normal cells. This selective generation of ROS in cancer cells distinguishes SC-III3 from other small molecules that affect ROS levels, such as paclitaxel and doxorubicin, which is suggestive of a selective mechanism of inhibition of HepG2 by SC-III3 [38, 39]. NAC pretreatment almost completely block SC-III3-induced p-H2AX expression and DNA damage response pathways, suggesting that SC-III3-induced DNA damage was ROS dependent. Furthermore, SC-III3-induced HepG2 cells S phase arrest and cell viability decrease were also reversed by NAC pretreatment. Collectively, these data demonstrated that SC-III3 mediated DNA damage and subsequent S phase arrest through inducing intracellular ROS generation.

## Conclusions

In summary, we demonstrated that cell growth inhibition and S phase arrest could occur in HepG2 cells exposed to SC-III3, and that these changes were mediated by ROS generation, which in turn activated ATM-Chk1/2-Cdc25A-Cdk2 pathway joined by ATM-p53-p21 branch, leading to the S phase arrest. Furthermore, SC-III3 also inhibited tumor growth of HepG2 xenograft mouse model. These data provided new insights into the anti-cancer mechanisms of SC-III3 and related compounds, which may be helpful to the development of SC-III3 into a promising therapeutic agent against hepatocellular carcinoma cancer.
